# Simultaneous tracking of movement and gene expression in multiple Drosophila melanogaster flies using GFP and DsRED fluorescent reporter transgenes

**DOI:** 10.1186/1756-0500-2-58

**Published:** 2009-04-17

**Authors:** Dhruv Grover, Junsheng Yang, Daniel Ford, Simon Tavaré, John Tower

**Affiliations:** 1 Molecular and Computational Biology Program, Department of Biological Sciences, University of Southern California, Los Angeles, California 90089-2910, USA; 2 Department of Applied Mathematics and Theoretical Physics, University of Cambridge, Cambridge CB3 0WA, UK

## Abstract

**Background:**

Fluorescent proteins such as GFP (Green Fluorescent Protein) and DsRED (Discosoma sp.Red Fluorescent Protein) are often used as reporter molecules for transgene expression in Drosophila and other species. We have recently reported methods that allow simultaneous tracking of animal movement and GFP expression in real time, however the assay was limited to single animals and a single transgene. Numerous studies would be facilitated by methods that allow for assay of multiple animals and multiple transgenes.

**Findings:**

Here we report an improved fly video tracking system that allows multiple transgenic flies to be tracked simultaneously using visible light, GFP fluorescence and DsRED fluorescence. The movement of multiple flies could be accurately tracked at real time rates, while simultaneously assaying the expression level of two different transgenes marked with GFP and DsRED. The individual flies could be accurately tracked and distinguished even during periods when transgene fluorescence was undetected. For example, characteristic patterns of *hsp70 *and *hsp22 *transgene induction could be simultaneously quantified and correlated with animal movement in aging flies, and as groups of flies died due to dessication/starvation.

**Conclusion:**

The improved methods allow for more efficient assay of the correlation between gene expression, behavior, aging and mortality: multiple animals can be assayed with simultaneous quantification of multiple transgenes using GFP and DsRED fluorescence. These methods should allow for increased flexibility in experimental designs. For example, in the future it should be possible to use gene expression levels to predict remaining life span more accurately, and to quantify gene expression changes caused by interactions between animals in real time.

## Background

The use of autofluorescent proteins such as Green Fluorescent Protein (GFP) has caused a revolution in biology and the study of gene expression [1,2]. Because the proteins require no added substrate in order to fluoresce, they require no invasive procedures for their assay, and serve as ideal reporters for the expression of transgenes in cells and whole animals. In addition, protein activity, protein-protein interactions, and protein sub-cellular locations can be assayed using fluorescence [3,4].

Video tracking is increasingly being used in studies of movement, behavior, and aging across species [5-8]. Examples include detailed studies of movement behavior in Drosophila[9,10] and *C. elegans *[11]. We have recently reported a video tracking system that allows tissue-specific GFP expression to be quantified and correlated with 3D animal movement in real time. In this way specific temporal patterns of gene expression can be correlated with temporal patterns of animal activity, behavior and mortality. For example, these procedures revealed that the oxidative stress-response genes *hsp70 *and *hsp22 *are induced during fly aging in circadian patterns (24 hr and 18 hr periods, respectively), and spike in the hours preceding and overlapping the death of the animal [12].

Numerous studies would be facilitated by methods that allow for assay of multiple animals and multiple transgenes. For example, interactions between animals, such as mating in Drosophila[13-15], are associated with changes in gene expression. Here we report the development of methods that allow for assay of the expression of multiple transgenes along with movement and behavior in groups of animals.

## Methods

### Drosophila cultureand strains

Drosophila* melanogaster *strains were cultured on yeast/cornmeal/agar/dextrose media [16], and flies of defined age were generated as previously described [17]. The *3xP3*-GFP [I4] and *3xP3*-GFP [M1] strains contain a construct in which eGFP expression is driven by an artificial promoter with three binding sites for the Eyeless transcription factor, and were provided by E. Wimmer [18]. The construction and characterization of the *hsp70 *and *hsp22 *fluorescent reporter constructs and transgenic strains has been recently described [19]: briefly, the *hsp22*-GFP and *hsp22*-DsRED constructs contain the *hsp22 *promoter sequences from -314 to +10 driving expression of eGFP and DsRED proteins, respectively, while the corresponding *hsp70 *reporter constructs contain *hsp70 *promoter sequences from -194 to +10. For certain experiments a doxycycline-regulated promoter was used to drive expression of DsRED: the reporter strain was Tet-on-DsRED [26B] (genotype *y ac w; p{dsRED}(3) [26B]*), and the construction and characterization of the Tet-on-DsRED strains will be described in detail elsewhere (N. Hoe, D. Ford and J.Tower, manuscript in preparation). To produce high-level, tissue-general expression of DsRED, the Tet-on-DsRED [26B] reporter strain was crossed to the "TO-tubulin" driver strain (genotype *w[1118]; p{UAS-rtTA-M2-alt} [901]/CyO; p{tubP-GAL4} [LL7]/TM6B*) [17], to generate progeny flies in which the *tubulin*gene regulatory sequences drive expression of GAL4, the GAL4 drives expression of rtTA-M2-alt, and rtTA-M2-alt then drives expression of Tet-on-DsRED, in a doxycycline-dependent manner. The flies containing these constructs were cultured on food supplemented with doxycycline for one week [17] to cause expression of DsRED prior to tracking assay.

### Multiple fly GFP and DsRED tracking

Tracking of multiple flies using GFP and DsRED reporter transgenes was accomplished by integrating procedures recently developed for tracking single flies using GFP [12] and for tracking multiple flies using visible light [20]. The flies were placed in standard 25 × 75 mm polyethylene culture vials containing 5 ml of solid food at the bottom and stoppered with cotton at the top. For certain experiments flies were placed in an empty vial to cause death by dessication/starvation. The vial was placed in the center of the circular camera rig, 70 cm in diameter. Six calibrated and synchronized Flea digital cameras (Point Grey) were mounted on the camera rig, facing downwards at a distance of 15 cm from the vial. Each camera was fitted with a megapixel 8 mm fixed focal lens (Edmund Optics). To detect GFP expression in flies, similar to protocols in our previous paper [12], the camera setup was modified with a blue-wavelength excitation light source and a visible range barrier filter. The light source was a 5W Luxeon V star 450 nm endura bright royal blue lambertian LED (Optotech, Cat # OT16-5100-RB). The LED was powered with a xitanium 700 mA LED driver (Optotech, AC converter Cat # OTMI-0060). A 515 nm barrier filter (Edmund Optics, Cat # NT39-417) was placed between the sensor and the camera lens to detect GFP expression. Similarly, to detect DsRED expression in flies a green-wavelength excitation light source and visible range barrier filter were added to the camera setup. The light source was a 5W Luxeon V star 550 nm endura bright green lambertian LED (Optotech, Cat # OT16-5100-G). The driver used to power the LED was identical to that used to power the blue-wavelength LED. A 585 nm barrier filter (Edmund Optics, Cat # NT39-417) was placed between the sensor and the camera lens to detect DsRED expression.

In order for the tracking algorithm [20] to keep track of multiple flies expressing GFP and DsRED, the tracking algorithm was modified to construct each three-dimensional fly visual hull using camera views that can detect only GFP/DsRED fluorescence due to the filters, plus camera views capable of detecting flies under the visible blue and green input light.

Prior to the fluorescence tracking assays, flies were cultured to the indicated age by transfer to new food every other day, under a 12 hr/12 hr light/dark cycle. The fluorescence tracking assays were conducted in a dark room where the only sources of illumination were the blue and/or green LEDs, and all experiments were initiated at the same time of day (4 PM). Detailed protocols are available for download from the laboratory website .

## Results and discussion

We have previously reported methods that allow for simultaneous real time tracking of movement and behavior in groups of flies using multiple cameras in visible light [20]. For visible light tracking, the flies are illuminated with white light, and their position in three-dimensional space for each frame is derived from fly silhouettes detected in multiple video camera views. To keep track of flies between frames and in periods of occlusion, an extended Kalman filter (EKF) tracking approach is used to estimate the current three-dimensional position and orientation of each fly in real time. This method allows individuals in a group to be tracked accurately even after occlusion, ie, an overlap in their trajectories or paths of movement. We have also recently reported methods that allow for the simultaneous tracking of movement and GFP fluorescence in a single fly {Grover, 2008 #4844}. For the single-fly GFP tracking, the fly is illuminated with blue input light, and the output green GFP fluorescence is detected using video cameras that are fitted with barrier filters to block out all but the GFP signal. The GFP fluorescence is used to track the movement of the fly, and the intensity of GFP fluorescence provides a metric for the level of transgene expression. In this way temporal patterns of movement and behavior can be correlated with specific temporal patterns of gene expression.

To allow for the simultaneous tracking of the movement of multiple flies along with the assay of both GFP and DsRED reporter transgenes, the methods for visible light fly tracking were combined with those for tracking flies with fluorescence. The fly vial was placed in the dark, and the flies were illuminated using LED lights, either blue (the input for excitation of GFP), green (the input for DsRED), or both. Two (or three, depending upon the experiment) cameras were used to detect flies under visible light, which is the input blue and/or green light; two (or three) cameras were fitted with barrier filters to allow detection of only GFP fluorescence; and two (or three) cameras were fitted with filters to detect only DsRED fluorescence. The tracking algorithm was modified to use the information from all six cameras to track the position and movement of each fly in the group, while the information from the filtered cameras was used to quantify simultaneously the intensity of GFP or DsRED fluorescence emitted from each individual fly.

**Figure 1:**
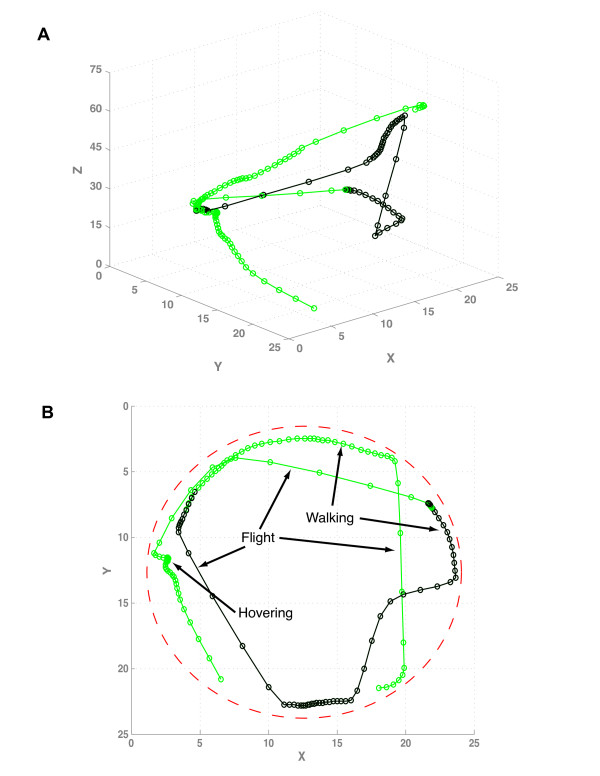
**Trajectory of a fly in 3D space**. A young (one day old) *3xP3*-GFP fly was tracked for 30 seconds using visible (blue) light and GFP fluorescence, including a 10 second interval where the GFP fluorescence data was not utilized (indicated in dark green). Circles indicate the location of the fly at each 1/4 second (250 ms). (A) Trajectory plotted in three dimensions. (B) Trajectory plotted in two dimensions, top-down view. The location of the wall of the vial is indicated with red dashes. Arrows and labels indicate regions of the trajectory that represent flight and hovering, as well as regions that most likely represent walking.

Several types of experiments were conducted to test that the system works as designed. First, an experiment was performed to test if the system can accurately track flies using both visible and fluorescent light. A single male fly that contains the *3xP3*-GFP reporter was assayed. The *3xP3*-GFP reporter contains a promoter with multiple binding sites for the Eyeless (Pax6 homolog) transcription factor, and is expressed in adult retinal tissue [18], with a circadian variation in expression (12 hr period) that follows closely the fly's daily activity patterns [12]. The fly was illuminated with blue light, and three cameras were used to detect the fly in visible (blue) light, and three cameras were used to detect the GFP fluorescence. Information from all six cameras was then used by the EKF to estimate the position and orientation of the fly over a period of time. The fly was tracked for 30 seconds, and the 3D trajectory through space is diagrammed (Figure 1). For a 10 second interval the GFP fluorescence was not input into the tracking algorithm (period indicated in dark green in Figure 1), and the system was able to track the fly accurately during this period using the information from the visible-light cameras, as indicated by the uninterrupted trajectory that was obtained. Therefore, the system is capable of tracking fly movement even during periods when fluorescence is not detected. This capability should be useful in the future for study of genes that turn on or off completely during the course of an experiment.

The tracking system detects all fly movement, which includes flight, walking and jumping, and a combination of these movements produces the measured fly trajectories. Based on the known characteristics of fly movements, it is possible in several instances to infer what behavior is represented by specific intervals of the trajectories. For example, the fly jump response has been analyzed in detail [21,22]: the maximum jump duration is ~250 ms, and the maximum distance a fly can travel by jump alone is ~30 mm, as assayed using wingless flies [23]. Therefore, movement of the fly through space for a greater time or distance must include flight. Because the dimensions and spatial position of the vial are known, such information can be used to help determine the specific type of movement represented by a particular portion of the trajectory. For example, as indicated in Figure 1B, there are several intervals where the fly moved rapidly through several centimeters of space between the walls of the vial, and therefore this movement must represent free flight. In contrast, there are several regions of the trajectory where the fly moved at a relatively slow speed immediately adjacent to the vial wall, and this movement is likely to represent walking. Drosophila are able to hover [24], and a position is indicated where the fly maintained the same location in the internal space of the vial for a sustained period that likely represents a hovering event (Figure 1B). To distinguish unambiguously between walking and hovering at locations near the vial edge would likely require additional information, such as whether the fly is moving its wings. We have recently reported methods that allow wing movement to be quantified (T. Goldman, E. Peebles, DG, ST, and M. Arbeitman, in preparation), however wing movement was not assayed in the experiments presented here.

**Figure 2:**
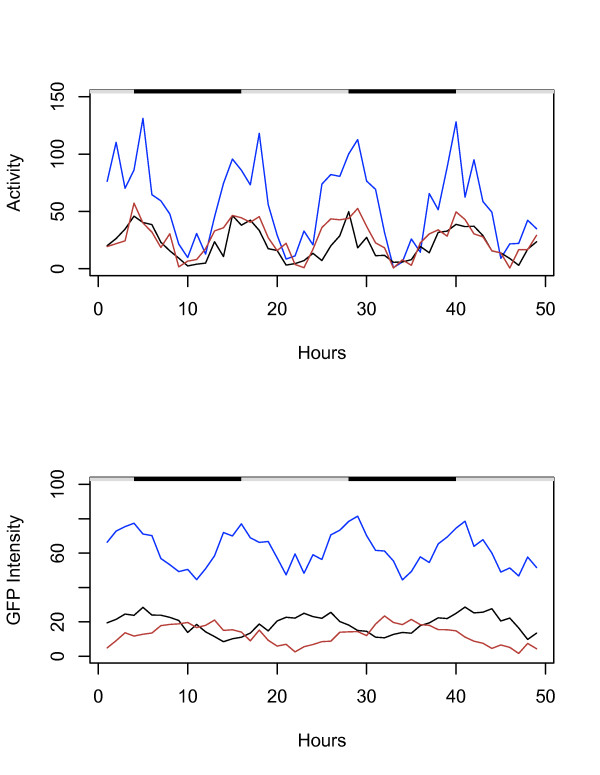
**Tracking movement and gene expression of multiple flies using GFP**. Multiple male flies were placed in a vial and tracked simultaneously for 48 hours. Black/white bars indicate the light/dark cycle the flies were cultured under prior to the beginning of the experiment. Activity is expressed as distance moved per hour (cm/hr), and GFP intensity is expressed as average pixel intensity per hour. *3xP3*-GFP, transgenic strain I4, 1 day old (Blue). *hsp70*-GFP, transgenic strain 2MI4, 1 month old (Brown). *hsp22*-GFP, transgenic strain 1MI1, 1 month old (Black).

To determine if the movement of multiple flies could be tracked while simultaneously quantifying gene expression in each fly using GFP, three flies each containing a different type of GFP reporter transgene were assayed. A one-day-old *3xP3*-GFP fly along with a one-month-old *hsp70*-GFP fly and a one-month-old *hsp22*-GFP fly were tracked for 48 hours, using three cameras for visible light and three cameras for GFP fluorescence (Figure 2). The system accurately tracked the 12 hour activity period for each fly (Figure 2, upper panel), as well as the characteristic circadian patterns of GFP expression expected for each fly (Figure 2, lower panel). The *3xP3*-GFP reporter expression (indicated in blue) peaked every 12 hours, corresponding to the flies' daily activity patterns, while the *hsp70*-GFP reporter (indicated in brown) was expressed in a 24 hour period, and the *hsp22*-GFP reporter was expressed with an 18 hour period (indicated in black).

**Figure 3:**
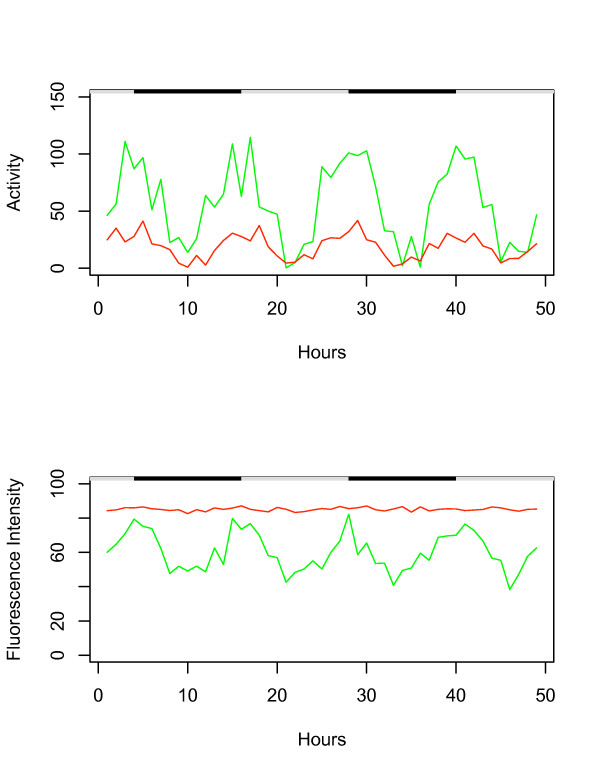
**Tracking movement and gene expression of multiple flies using GFP and DsRED**. Two male flies were placed in a vial and tracked simultaneously for 48 hours. Black/white bars indicate the light/dark cycle the flies were cultured under prior to the beginning of the experiment. Activity is expressed as distance moved per hour and fluorescence intensity is expressed as average pixel intensity per hour. *3xP3*-GFP, transgenic strain I4, 7 days old (Green). Tet-on-DsRED, transgenic strain (2)26B, 1 month old (Red).

To determine if multiple flies could be tracked along with assay of both GFP and DsRED fluorescence, two flies were tracked for 48 hours (Figure 3): one fly contained the *3xP3*-GFP reporter (indicated in green), and a second fly contained a doxycyline-regulated promoter driving expression of DsRED (Tet-on-DsRED; indicated in red). In the Tet-on-DsRED fly, DsRED expression is ultimately being driven by the regulatory sequences of the tissue-general *tubulin *gene (see Methods). The system accurately tracked the movement and 12 hour activity period for each fly (Figure 3, upper panel), as well as the expected 12 hour period of GFP expression pattern for the *3xP3*-GFP fly, and the high level and constant expression of DsRED for the Tet-on-DsRED fly (Figure 3, lower panel).

The blue and green input light used to excite the GFP and DsRED reporters, respectively, is in the range visible to the fly [25]. Drosophila maintains relatively normal circadian rhythms of activity under constant light illumination, however the period of activity lengthens slightly after several days, and more intense light levels can cause arrhythmicity [26]. Fly activity levels and circadian rhythms were not obviously affected by the relatively low-level and brief (48 hours) constant illumination with blue and/or green light employed here; however, whether constant illumination with blue and/or green light might have more subtle effects on fly behaviors remains to be determined.

**Figure 4:**
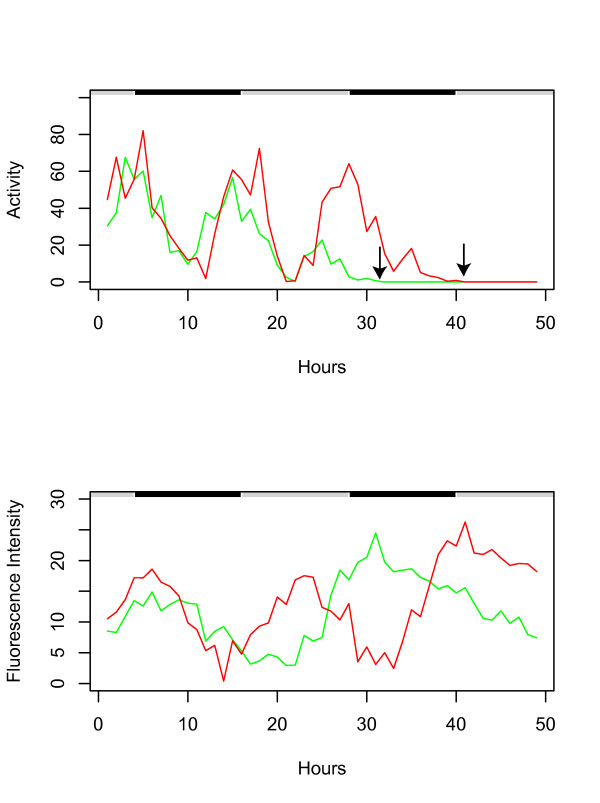
**GFP and DsRED reporter expression in separate flies dying from dessication/starvation**. Two male flies were placed in an empty vial and tracked for 48 hours. Black/white bars indicate the light/dark cycle the flies were cultured under prior to the beginning of the experiment. Activity is expressed as distance moved per hour and fluorescence intensity is expressed as average pixel intensity per hour. Arrows indicate the last time point at which the animals displayed spontaneous movement; these animals were scored as dead at the end of the experiment based on lack of movement in response to stimulation. hsp22-DsRED, transgenic strain 1MI1, 20 days old (Red), hsp70-GFP, transgenic strain 2MI4, 20 days old (Green).

**Figure 5:**
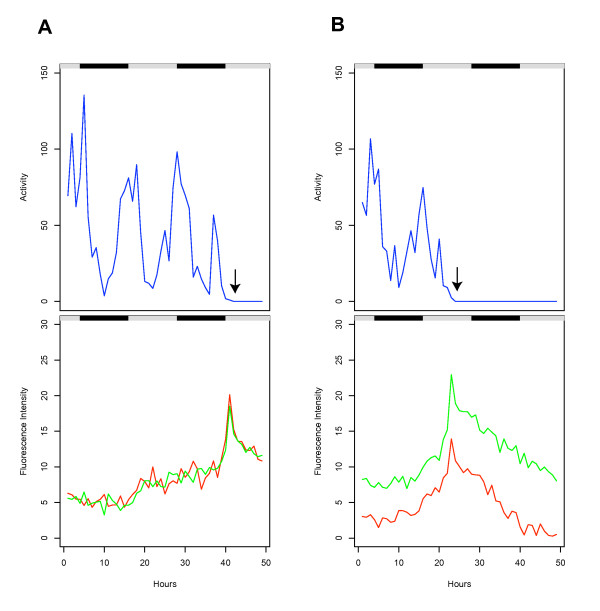
**GFP and DsRED reporter expression in the same fly dying from dessication/starvation**. Two male flies were placed in an empty vial and tracked for 48 hours. Black/white bars indicate the light/dark cycle the flies were cultured under prior to the beginning of the experiment. Activity is expressed as distance moved per hour (cm/hr, indicated in blue) and fluorescence intensity is expressed as average pixel intensity per hour: GFP fluorescence is plotted in green and DsRED fluorescence is plotted in red. Arrows indicate the last time point at which the animals displayed spontaneous movement; these animals were scored as dead at the end of the experiment based on lack of movement in response to stimulation. One fly contained both the hsp70-GFP and hsp22-DsRED transgenes, genotype w[1118]; p{hsp22-DsRED} [1MI1]/CyO; p{hsp70-GFP} [1MI2]/TM3 Sb (A). The second fly contained both the hsp22-GFP and hsp70-DsRED transgenes, genotype w[1118]; p{hsp70-DsRED} [1MI2]/CyO; p{hsp22-GFP} [1MI1]/TM3 Sb (B).

Finally, to test if the expression of multiple transgenes could be correlated with behavior in multiple flies, two flies were assayed for 48 hours in an empty vial, during which time they died from dessication/starvation, as expected (Figure 4). One fly contained the *hsp70*-GFP reporter (indicated in green), while the other fly contained the *hsp22*-DsRED reporter (indicated in red). The system accurately tracked the movement of the flies (Figure 4, upper panel), including the 12-hour period of activity levels, and the cessation of movement indicative of death (indicated by black arrows). The circadian variation in the level of *hsp70 *and *hsp22 *transgene expression characteristic of older flies was detected, as was the spike in expression of each transgene in the hours preceding and overlapping the death of the animal (Figure 4, lower panel). Finally, not only was it possible to track *hsp70*-GFP and *hsp22*-DsRED expression simultaneously in two different flies, it was also possible to track and quantify accurately the GFP and DsRED reporters linked to *hsp70 *and *hsp22 *transgenes at the same time in a fly that contained both transgenes: One fly contained both the *hsp70*-GFP and *hsp22*-DsRED transgenes (Figure 5A), while the other fly contained both the *hsp22*-GFP and *hsp70*-DsRED transgenes (Figure 5B). The flies were tracked while dying from dessication/starvation. Because these were young flies, the level of hsp reporter expression is initially low, and then spikes dramatically preceding and overlapping the death of the animal (indicated with black arrows). These results demonstrate that two different transgenes can be accurately quantified in a single fly and correlated with behavior and mortality.

An important consideration in the simultaneous tracking of movement and gene expression in groups of animals is ability of the system to distinguish the individual animals accurately and continuously. For these experiments, reporter transgenes were chosen that have very different and characteristic circadian patterns of reporter expression: the *3xP3*-GFP reporter has a 12 hour period, the *hsp70 *reporters have a 24 hour period, the *hsp22 *reporters have an 18 hour period, and the Tet-on reporter combined with the *tubulin *gene driver has high-level and relatively constant expression. When these flies were tracked together in groups, each of these characteristic patterns was accurately and continuously observed across the multi-hour tracking sessions. If the system were to fail to continually distinguish two flies, for example due to the flies moving close together or colliding (occlusion), one would expect an abrupt switch in the measured fluorescence from one pattern to another. Notably, no such event was ever detected in our experiments, indicating that the system is able to distinguish the individual flies in a group accurately and continuously, despite their complex and overlapping trajectories. Previous control experiments demonstrated that the tracking algorithm is able to track robustly up to 10 flies in a vial simultaneously without errors, whereas increasing the number of flies in the vial beyond 10 reduces tracking efficiency [20].

## Conclusion

The improved fly tracking system utilizes six calibrated and synchronized video cameras, and allows for multiple transgenic flies to be tracked simultaneously using visible light, GFP fluorescence and DsRED fluorescence. The movement of multiple flies could be accurately tracked at real time rates, while simultaneously assaying the expression level of two different transgenes marked with GFP and DsRED. Individual flies within a group could be accurately and continually distinguished, even during periods of occlusion (over-lapping trajectories) and during periods when transgene fluorescence from a particular fly was undetected. These improved methods allow for more efficient assay of the correlations between gene expression, behavior, aging, and mortality, and should allow for increased flexibility in experimental designs. For example, in the future it should be possible to use *hsp *gene expression levels to predict the remaining life span of individuals [19] more accurately, and to quantify gene expression changes caused by interactions between animals in real time.

## Competing interests

The authors declare that they have no competing interests.

## Authors' contributions

JY and DF generated and characterized the transgenic fly strains, DG and ST devised the tracking methods and algorithms and DG conducted all tracking experiments, JT designed the overall study, and DG and JT wrote the paper. The final manuscript has been read and approved by all authors.

## References

[B1] Miyawaki A (2008). Green fluorescent protein glows gold. Cell.

[B2] Shaner NC, Patterson GH, Davidson MW (2007). Advances in fluorescent protein technology. J Cell Sci.

[B3] Fernandez-Suarez M, Ting AY (2008). Fluorescent probes for super-resolution imaging in living cells. Nat Rev Mol Cell Biol.

[B4] VanEngelenburg SB, Palmer AE (2008). Fluorescent biosensors of protein function. Curr Opin Chem Biol.

[B5] Fontaine E, Lentink D, Kranenbarg S, Mυller UK, van Leeuwen JL, Barr AH, Burdick JW (2008). Automated visual tracking for studying the ontogeny of zebrafish swimming. J Exp Biol.

[B6] Ramot D, Johnson BE, Berry TL, Carnell L, Goodman MB (2008). The Parallel Worm Tracker: a platform for measuring average speed and drug-induced paralysis in nematodes. PLoS ONE.

[B7] Rougier C, Meunier J, St-Arnaud A, Rousseau J (2006). Monocular 3D head tracking to detect falls of elderly people. Conf Proc IEEE Eng Med Biol Soc.

[B8] Rutz C, Bluff LA (2008). Animal-borne imaging takes wing, or the dawn of 'wildlife video-tracking'. Trends Ecol Evol.

[B9] Valente D, Golani I, Mitra PP (2007). Analysis of the Trajectory of *Drosophila melanogaster *in a Circular Open Field Arena. PLoS ONE.

[B10] Fry SN, Rohrseitz N, Straw AD, Dickinson MH (2008). TrackFly: virtual reality for a behavioral system analysis in free-flying fruit flies. J Neurosci Methods.

[B11] Tsechpenakis G, Bianchi L, Metaxas D, Driscoll M (2008). A novel computational approach for simultaneous tracking and feature extraction of *C. elegans *populations in fluid environments. IEEE Trans Biomed Eng.

[B12] Grover D, Yang J, Tavare S, Tower J (2008). Simultaneous tracking of fly movement and gene expression using GFP. BMC Biotechnol.

[B13] McGraw LA, Clark AG, Wolfner MF (2008). Post-mating gene expression profiles of female *Drosophila melanogaster *in response to time and to four male accessory gland proteins. Genetics.

[B14] Kapelnikov A, Zelinger E, Gottlieb Y, Rhrissorrakrai K, Gunsalus KC, Heifetz Y (2008). Mating induces an immune response and developmental switch in the *Drosophila *oviduct. Proc Natl Acad Sci USA.

[B15] Krupp JJ, Kent C, Billeter JC, Azanchi R, So AK, Schonfeld JA, Smith BP, Lucas C, Levine JD (2008). Social experience modifies pheromone expression and mating behavior in male *Drosophila melanogaster*. Curr Biol.

[B16] Ren C, Webster P, Finkel SE, Tower J (2007). Increased internal and external bacterial load during *Drosophila *aging without life-span trade-off. Cell Metab.

[B17] Ford D, Hoe N, Landis GN, Tozer K, Luu A, Bhole D, Badrinath A, Tower J (2007). Alteration of *Drosophila *life span using conditional, tissue-specific expression of transgenes triggered by doxycyline or RU486/Mifepristone. Exp Gerontol.

[B18] Horn C, Jaunich B, Wimmer EA (2000). Highly sensitive, fluorescent transformation marker for *Drosophila *transgenesis. Dev Genes Evol.

[B19] Yang J, Tower J (2009). Expression of *hsp22 *and *hsp70 *transgenes is partially predictive of *Drosophila *survival under normal and stress conditions. J Gerontol: Biol Sci.

[B20] Grover D, Tower J, Tavare S (2008). O fly, where art thou?. J R Soc Interface.

[B21] Kaplan WD, Trout WE (1974). Genetic manipulation of an abnormal jump response in *Drosophila*. Genetics.

[B22] Card G, Dickinson MH (2008). Visually mediated motor planning in the escape response of *Drosophila*. Curr Biol.

[B23] Zumstein N, Forman O, Nongthomba U, Sparrow JC, Elliott CJ (2004). Distance and force production during jumping in wild-type and mutant *Drosophila *melanogaster. J Exp Biol.

[B24] Homyk T (1977). Behavioral mutants of *Drosophila Melanogaster*. II. Behavioral Analysis and Focus Mapping. Genetics.

[B25] Montell C (1999). Visual transduction in *Drosophila*. Annu Rev Cell Dev Biol.

[B26] Konopka RJ, Pittendrigh C, Orr D (2007). Reciprocal behaviour associated with altered homeostasis and photosensitivity of *Drosophila *clock mutants. J Neurogenet.

